# Electroacupuncture reduces chronic itch *via* cannabinoid CB1 receptors in the ventrolateral periaqueductal gray

**DOI:** 10.3389/fphar.2022.931600

**Published:** 2022-09-05

**Authors:** Wen-Qiang Ge, Ou-Yang Zhan-Mu, Chao Chen, Hong Zhang, Xiao-Yu Wang, Xin Liu, Li Li, Yu-Ye Lan, Chen-Nan Li, Jia-Can Sun, Run-Lin Shi, Zi-Yue Dou, Hui-Lin Pan, Hong-Ping Li, Xiang-Hong Jing, Man Li

**Affiliations:** ^1^ Department of Neurobiology, School of Basic Medicine, Tongji Medical College of Huazhong University of Science and Technology, Wuhan, China; ^2^ Institute of Acupuncture and Moxibustion, China Academy of Chinese Medical Sciences (CACMS), Beijing, China; ^3^ Department of Dermatology, Union Hospital, Tongji Medical College, Huazhong University of Science and Technology, Wuhan, China; ^4^ Department of Anesthesiology and Perioperative Medicine, The University of Texas MD Anderson Cancer Center, Houston, TX, United States

**Keywords:** Acupuncture, chronic itch, CB1 receptors, PAG, glutamate, GABA

## Abstract

Chronic itch severely reduces the quality of life of patients. Electroacupuncture (EA) is widely used to treat chronic itch. However, the underlying mechanism of this therapeutic action of EA is largely unknown. Cannabinoid CB1 receptors in the ventrolateral periaqueductal gray (vlPAG) mediate the analgesic effect of EA. Using a dry skin-induced itch model in mice, we determined whether EA treatment reduces chronic itch via CB1 receptors in the vlPAG. We showed that the optimal inhibitory effect of EA on chronic itch was achieved at the high frequency and high intensity (100 Hz and 3 mA) at “Quchi” (LI11) and “Hegu” (LI14) acupoints, which are located in the same spinal dermatome as the cervical skin lesions. EA reversed the increased expression of CB1 receptors in the vlPAG and decreased the concentration of 5-hydroxytryptamine (5-HT) in the medulla oblongata and the expression of gastrin-releasing peptide receptors (GRPR) in the cervical spinal cord. Furthermore, knockout of CB1 receptors on GABAergic neurons in the vlPAG attenuated scratching behavior and the 5-HT concentration in the medulla oblongata. In contrast, knockout of CB1 receptors on glutamatergic neurons in the vlPAG blocked the antipruritic effects of EA and the inhibitory effect of EA on the 5-HT concentration in the medulla oblongata. Our findings suggest that EA treatment reduces chronic itch by activation of CB1 receptors on glutamatergic neurons and inhibition of CB1 receptors on GABAergic neurons in the vlPAG, thereby inhibiting the 5-HT release from the medulla oblongata to GRPR-expressing neurons in the spinal cord. Our findings suggest that EA attenuates chronic itch *via* activating CB1 receptors expressed on glutamatergic neurons and downregulating CB1 receptors on GABAergic neurons in the vlPAG, leading to the reduction in 5-HT release in the rostroventral medulla and GRPR signaling in the spinal cord. Our study not only advances our understanding of the mechanisms of the therapeutic effect of EA on chronic itch but also guides the selection of optimal parameters and acupoints of EA for treating chronic itch.

## Introduction

Chronic itch (symptoms last >6 weeks) is associated with many systemic diseases and seriously reduces the quality of life of patients (Metz et al., 2011). Scratching behavior can lead to a greater area of itch (Sharma et al., 2006) and skin tissue damage (Akiyama and Carstens, 2013; LaMotte et al., 2014). Current treatment with Western medicine is not satisfactory (Yosipovitch et al., 2003). Electroacupuncture (EA), an important part of traditional medical practice in China, is becoming increasingly popular as an alternative treatment option for chronic itch. Previous experimental and clinical studies have shown that EA can effectively reduce chronic itch caused by psoriasis, atopic dermatities, urticaria, uremic pruritus, and other chronic allergic skin diseases (Duo, 1987; Kim et al., 2010; Jung et al., 2014; Wang et al., 2019; Zhang et al., 2020a; Zhang et al., 2020b). Although EA is effective in managing chronic itch, the mechanism underlying the anti-pruritic effect of EA is still largely unknown.

Increased activity of both cannabinoid CB1 receptors and cannabinoid CB2 receptors can reduce itch (Schlosburg et al., 2011; Odan et al., 2012). The analgesic and antipruritic effects were predominately mediated through CB1 receptors in the central nervous system (Schlosburg et al., 2011; Bilir et al., 2018; Yuan et al., 2018). The CB1 receptors antagonists, such as rimonabant, elicit a dose-dependent increase in scratching behavior in mice (Darmani and Pandya, 2000), and activation of CB1 receptors reduces the scratching response (Schlosburg et al., 2009). However, the loci of CB1 receptors expressed in the central nervous system that are involved in the anti-pruritic effect of EA is unclear.

Chronic itch is controlled by the itch signaling pathway in the brainstem (Akiyama et al., 2015; Bourane et al., 2015). It has been shown that 5-hydroxytryptamine (5-HT)-expressing neurons, which are involved in the brainstem descending analgesia system, can aggravate pruritus by activating 5-HT1A receptors on itch-specific, gastrin-releasing peptide receptors (GRPR) neurons in the spinal cord and central sensitization of itch (Zhao et al., 2014). The release of 5-HT from the medulla oblongata, especially the rostroventral medulla (RVM), to the spinal cord is regulated by glutamatergic and GABAergic neurons in the periaqueductal gray (PAG) (Maione et al., 1998; Yuan et al., 2018). Moreover, activation of GABAergic neurons or inhibition of glutamatergic neurons in the PAG results in attenuation of scratching in both acute and chronic itch animal models (Samineni et al., 2019). In addition, we have found that CB1 receptors on GABAergic, but not glutamatergic, neurons are involved in the EA effect on descending inhibitory control of 5-HT in the medulla oblongata in a chronic pain model (Yuan et al., 2018). Because pain and itching are closely related, we reasoned whether EA alleviates chronic itch behavior via CB1 receptors on GABAergic and glutamatergic neuron in the ventrolateral PAG (vlPAG).

In the present study, we first determined the most effective stimulation parameters of EA on chronic itch. Then, we determined whether EA reduces chronic itch via altering the 5-HT concentration in the medulla oblongata and the expression of GRPR in the spinal cord. Finally, we determined whether the effect of EA on chronic itch is mediated by CB1 receptors expressed in glutamatergic or GABAergic neurons in the vlPAG.

## Materials and method

### Animals

Cnr1flox/flox mice (CB1-flox) were used to induce CB1 conditional KO in the vlPAG using a AAV virus vector. GRPR-iCreERT2:Ai14 mice were used to study the effects of EA on GRPR. For the other experiments, C57BL/6J mice were used. All mice (male, 8–10 weeks of age, 18–21 g) were housed under a 12-h light/12-h dark cycle, with lights on at 7 a.m. and food and water provided ad libitum. Adult C57BL/6 mice were purchased from Beijing Weitong Lihua Experimental Animal Technology Co., Ltd. (Beijing, China). The Cnr1flox/flox mice were bought from the Cyagen Biosciences Laboratory (Nanjing, China). GRPR-iCreERT2 were kindly provided by Dr. Yan-Gang Sun (Institute of Neuroscience, Chinese Academy of Sciences). GRPR-iCreERT2:Ai14 mice were bred by Shanghai Model Organisms Center, Inc. (Shanghai, China).

### Induction of chronic dry skin itch in mice

The mouse model of chronic dry skin itch was established to observe itch behaviors following previously described procedures (Akiyama et al., 2012). Briefly, a piece of gauze (the area is approximately 15 × 15 mm^2^) infiltrated with a volume ratio (1:1) of acetone and diethyl ether was applied to the shaved portion of the mouse neck for 15 s and then another piece of gauze infiltrated by distilled water was used for another 30 s, twice a day for 9 consecutive days (Li et al., 2021), which was successful in producing chronic itch. The above acetone/diethyl ether and water treatment is designated as AEW. In the vehicle control group, the gauze infiltrated with distilled water was applied to the neck for 45 s.

### EA treatment

In the EA group, EA stimulation was performed on the left side of the mice, Hegu (LI4) and Quchi (LI11) or Xuehai (SP10) and Zusanli (ST36), from the first day of establishing the itch model. The EA frequency was 100 Hz, the intensity was 1 mA or 3 mA, and the wave width was 0.3 ms. EA was performed once every other day, and each treatment lasted for 30 min. A total of five treatments of EA were performed. The needles were inserted into the Hegu and Quchi points, respectively, 2–3 mm deep corresponding to that of humans (Wu et al., 2018). The Hegu point was located at the back of the hand, between the first and second metacarpals, and at the midpoint of the temporal side of the second metacarpal; when the elbow was fully curved, the mouse Quchi was located in the depression outside the elbow fold (Lim, 2010). Xuehai was located in the medial aspect of the thigh at a point 3 mm above the mediosuperior border of the patella with the knee flexed, on the bulge of the medial portion of the quadriceps femoris muscle. Zusanli was located at 1 mm lateral to the anterior tubercle of the tibia and 3 mm below the capitulum fibuae under the knee joint (Kan et al., 2018). The current was delivered through a Han’s acupoint nerve stimulator (LH202, China) that maintains a constant current output. During the EA or sham EA treatment, each mouse stayed in the homemade cloth and remained awake and painless. In the sham EA group, the acupuncture needles were only shallowly inserted into the LI4 and LI11 for 30 min without electrical stimulation. The vehicle control group and the chronic itch group mice were kept in homemade cloth for 30 min without any acupuncture treatment.

### Drug administration

AM251 [1-(2,4-dichlorophenyl)5-(4-iodophenyl)-4-methyl-N-1-piperidinyl-1H-pyrazole-3-carboxamide], a CB1 receptor antagonist (Sigma-Aldrich, Steinheim, Germany), was dissolved in the dimethyl sulfoxide (DMSO; Sigma-Aldrich) to achieve a concentration of 10 mg/ml. On the day of the experiment, 10 ml AM251 was dissolved in 100 ml of DMSO to achieve a final concentration of 1 mg/ml (Tjen et al., 2009). In the experiment using AM251, mice were randomly divided into the vehicle control group, AEW+DMSO group, AEW+AM251 group, EA+DMSO group and EA+AM251 group. The AEW + AM251 group and the EA + AM251 group were intraperitoneally injected with AM251 at a dose of 1 mg/kg 10 min before the EA (Tosun et al., 2015), once every other day, for a total of five injections. The mice in the AEW+DMSO group and the EA+DMSO group were intraperitoneally injected with the same volume of DMSO. Tamoxifen (Sigma, United States) was used to induce the expression of GRPR and was dissolved in corn oil (Sigma, United States) to achieve a concentration of 20 mg/ml.

### Virus injection

The injection procedures have been described in our previous studies (Zhu et al., 2019). In brief, mice were anesthetized with sodium pentobarbital (250 mg/kg, i. p.). The periosteum was gently removed from the exposed surface of the surgical area by cutting 1.5 cm lengthwise along the midline of the skull. Then a glass micropipette connected to a microsyringe (1 μL, Hamilton) was inserted into the target site. For knocking out the CB1 receptors on GABAergic neurons or glutamatergic neurons, AAV2/9-mDlx-Cre-WPRE-pA or AAV2/9-CamkII-Cre-WPRE-pA (Zhu et al., 2019)(Wuhan BrainVTA Scientific and Technical Corporation) was injected into the bilateral sides of the vlPAG of Cnr1flox/flox mice respectively, using a stereotactic holder at Bregma AP −4.8 mm mm, lateral 0.4 mm and depth 2.8 mm. Desired virus vectors (120 nL) were injected into the vlPAG at a rate of 50 nL per 60 s. The mice with the virus infection were allowed to recover for 21 days.

### Scratch behavior

To record the scratching behavior, all mice were sequentially placed in a separately Plexiglas recording arena with a transparent cover and continuously adapted for 3 days, 30 min each time (Nojima et al., 2003). We used a digital camcorder to record the mice’s behavior. Before the itch was induced, the hair of the mouse neck was shaved and randomly divided into the vehicle control group, a chronic itch model (AEW) and an EA treating group. The scratching behavior was recorded for 60 min as a baseline. The mice were continuously treated with acetone and diethyl ether twice a day for 9 consecutive days. When the itch behavior was tested on the 10th day, the mice were placed in the test chamber for 30 min, and then the scratching behavior was recorded for 60 min. During the process of recording, the experimenter left the lab room and kept the lab room quiet. The video was played back, and the number of scratches was counted by two experimenters who were blinded to the experiments groups. A series of one or more scratching movements by the hindlimb directed toward the neck dry skin area was regarded as one scratching bout, which ended when the mouse either bit/licked the toes or placed the hindlimb on the floor (Li et al., 2021).

### Western blotting

The procedures have been described in our previous studies (Li et al., 2021). Briefly, on the 10th day of dry skin induction, mice were anesthetized with sodium pentobarbital (250 mg/kg, i. p.), euthanized, and sacrificed by cervical dislocation. The brains were immediately excised and placed on ice to extract the vlPAG. These tissues (vlPAG) were homogenized in RIPA lysis buffer with 50 mg/ml (Beyotime Biotechnology, Nanjing, China). The homogenates were centrifuged at 12,000 × g for 15 min at 4°C. The concentrations of protein obtained from the supernatant were detected by the Enhanced BCA Protein Assay Kit (Beyotime Biotechnology, China). The protein (40 μg) was separated on an 10% glycine-SDS-PAGE gel and transferred onto a PVDF membrane (Millipore Immobilon-P, United States). Then, the transferred blot was blocked in Tris buffered saline (TBST) containing 5% skim milk powder at room temperature for 1 h. The membrane was placed in the following primary antibodies at 4°C overnight: rabbit anti-CB1 monoclonal antibody (1:1000, Cell Signaling Technology), rabbit anti-CB2 polyclonal antibody (1:500, Abcam), and mouse anti-GAPDH (0.5 μg/ml, Cloud-Clone Corp). In the next day, the membrane was removed from the primary antibody and incubated with the following secondary antibodies on a shaker at room temperature for 1 h: horseradish peroxidase-labeled goat anti-rabbit IgG (1:20000, Santa Cruz, United States) and horseradish peroxidase-labeled goat mouse IgG (1:20000, Santa Cruz, United States), 1 h. The signals were developed using Super Signal West Pico chemiluminescent substrate (Thermo Scientific, United States). The densitometric analysis of the protein band images was performed using ImageJ software (NIH, Bethesda, MD, United States).

### ISH and immunofluorescence

Mice were deeply anesthetized by intraperitoneal injection of sodium pentobarbital (250 mg/kg, i. p.), and sacrificed by perfusing through the ascending aorta with 37°C normal saline, followed by 4% paraformaldehyde (PFA) dissolved in 0.01 M phosphate-buffer solution (PBS, pH 7.4, 4°C) on the 10th day. These brains were extracted and preserved in diethyl pyrocarbonate (DEPC, sigma, United States)-PFA (4% PFA and 0.1% DEPC dissolved in 0.01 M phosphate-buffer solution) for 12 h. Then brains were transferred into DEPC-PBS (0.1% DEPC dissolved in 0.01 M phosphate-buffer solution) for 24 h. The brains were dehydrated by 20% and 30% sugar (20% or 30% sugar and 0.1% DEPC dissolved in 0.1 M phosphate-buffer solution). Then, these brains were dissected and cut into 30 μm thick transverse sections. *In situ* hybridization (ISH) was performed by using DIG detection kit (Boster, China). These sections were hybridized with digoxigenin-labeled GAD67 or VGLUT2 riboprobe according to the manufacturer’s instructions. After ISH histochemistry, the sections were incubated with a rabbit anti-CB1 polyclonal primary antibody (1:200, Abcam, United States) at 4°C for 48 h. A fluorescent secondary antibody (Donkey anti-rabbit IgG conjugated with Dynight 488, 1:400, Jackson Immuno Research) was added and incubated at 37°C for 1 h in the dark.

### Immunofluorescence

For c-Fos staining, mice were anaesthetized with sodium pentobarbital (250 mg/kg, i. p.) and sacrificed by perfusing through the ascending aorta with 37°C normal saline, followed by 4% PFA in 1.5 h after EA treatment on the 9th day. These brains were extracted and preserved in 4% PFA for 12 h. Then brains were transferred into 0.01M PBS for 24 h. The brains were dehydrated by 20% and 30% sugar and cut into 30 μm thick transverse sections. A rabbit anti-c-Fos polyclonal primary antibody (1:300, Abcam, United States) was incubated with the tissue section at 4°C for 24 h. A fluorescent secondary antibody (Donkey anti-rabbit IgG conjugated with Dynight 488, 1:400, Jackson Immuno Research) was added and incubated at 37°C for 1 h in the dark.

### Measurements of serotonin in the medulla oblongata

The procedures have been described in our previous studies (Yuan et al., 2018). The 5-HT concentration in the medulla oblongata was determined using fluorescence spectrophotometry. Briefly, after sacrificing the mouse, medulla tissue was immediately removed on the 10th day. After weighing, specimens were homogenized in 3 ml of cool acid butyl alcohol and centrifuged at 3,000 g for 5 min before collecting the supernatant. 2.5 ml of the supernatant was added into a 15-ml centrifuge tube, then added 5 ml of n-heptane and 1 ml of 0.1 mol/L hydrochloric acid and centrifuge at 3,000 g for 5 min at room temperature. About 0.5 ml of the aqueous phase was added into a 5 ml centrifuge tube, added 100 μL of 0.5% cysteine and 3 ml of 0.006% o-phthalaldehyde (OPT). After mixing thoroughly, the solution was placed into boiling water for 10 min. The fluorescence intensity of 5-HT was measured using a fluorescence spectrophotometer (F- 4500, Hitachi, Japan) at a wavelength of 365/480 mm.

### Statistical analysis

All data are expressed as mean ± standard error (S. E. M.). Statistical analysis was performed using Prism 9 software. Unpaired *t* test was used to compare the knockout of CB1 receptors. One-way ANOVA and Tukey’s multiple comparisons test was used in the analysis of 5-HT concentrations and expression of CB1 and CB2 receptors in the control group, AEW group, EA group and sham EA. Two-way ANOVA and Sidak’s multiple comparisons test were used in the analysis of scratching behavior and 5-HT concentrations in wild-type mice and CB1 receptor knockout mice. *p* < 0.05 was considered to be statistically significant.

## Results

### EA at 100 Hz and 3 mA optimally reduces itch-related behavior

**FIGURE 1:**
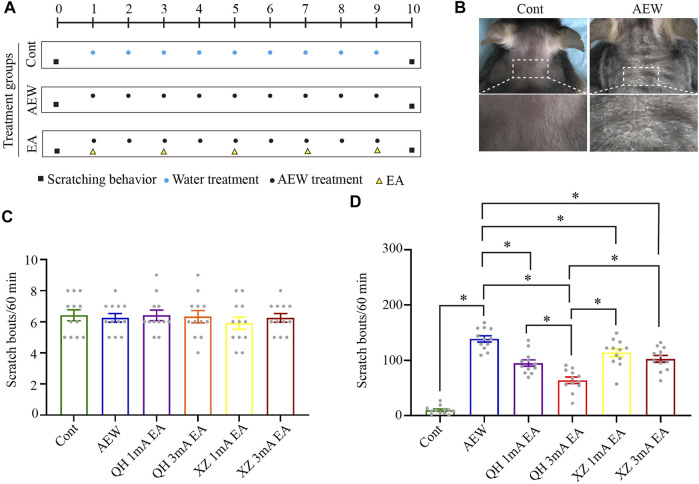
Effect of EA at different intensities and different acupuncture points on spontaneous scratching behavior. **(A)** A schematic showing the protocol of experiments and EA treatment. **(B)** The comparison of neck skin between the AEW group and control group. **(C)** The baseline scratch bouts in control, AEW, QH 1 mA EA, QH 3 mA EA, XZ 1 mA EA and XZ 3 mA EA groups. **(D)** The scratch bouts in control and AEW mice and the effects of QH 1 mA EA, QH 3 mA EA, XZ 1 mA EA and XZ 3 mA EA on scratch bouts in AEW mice. Data are expressed as means ± SEM (*n* = 12 mice in each group). *p < 0.05. QH, Quchi and Hegu; XZ, Xuehai and Zusanli.

Experimental and EA treatment protocols are shown in Figures 1A,B. On the day of chronic itch induction (AEW), different intensities and acupoints were tested in the four EA groups with the same frequency (100 Hz): Quchi and Hegu points with 1 mA (QH 1 mA) EA, Quchi and Hegu points 3 mA (QH 3 mA) EA, Xuehai and Zusanli points with 1 mA (XZ 1 mA) EA, and Xuehai and Zusanli points 3 mA (XZ 3 mA) EA. EA was administered for 30 min, once every other day for 5 times, starting from the 1st day after AEW administration. Before itch induction with acetone and ether, there was no significant difference in scratching behaviour among the groups (*p* > 0.05, Figure 1C). However, on the 10th day the number of scratching behaviour of AEW was significantly increased compared with the control group (*p* < 0.05, Figure 1D). Furthermore, the number of scratching behaviour of the four EA groups was significantly less than the AEW group (*p* < 0.05, Figure 1D). The number of scratching in QH 3 mA EA group was significantly less than that of QH 1 mA EA group (*p* < 0.05, Figure 1D), XZ 1 mA EA (*p* < 0.05, Figure 1D), and XZ 3 mA EA group (*p* < 0.05, Figure 1D). Therefore, EA using 100Hz, 3 mA at Quchi and Hegu points optimally reduces dry skin-induced chronic itch.

### EA reverses the up-regulation of CB1, but not CB2, receptors in the vlPAG in chronic itch

**FIGURE 2:**
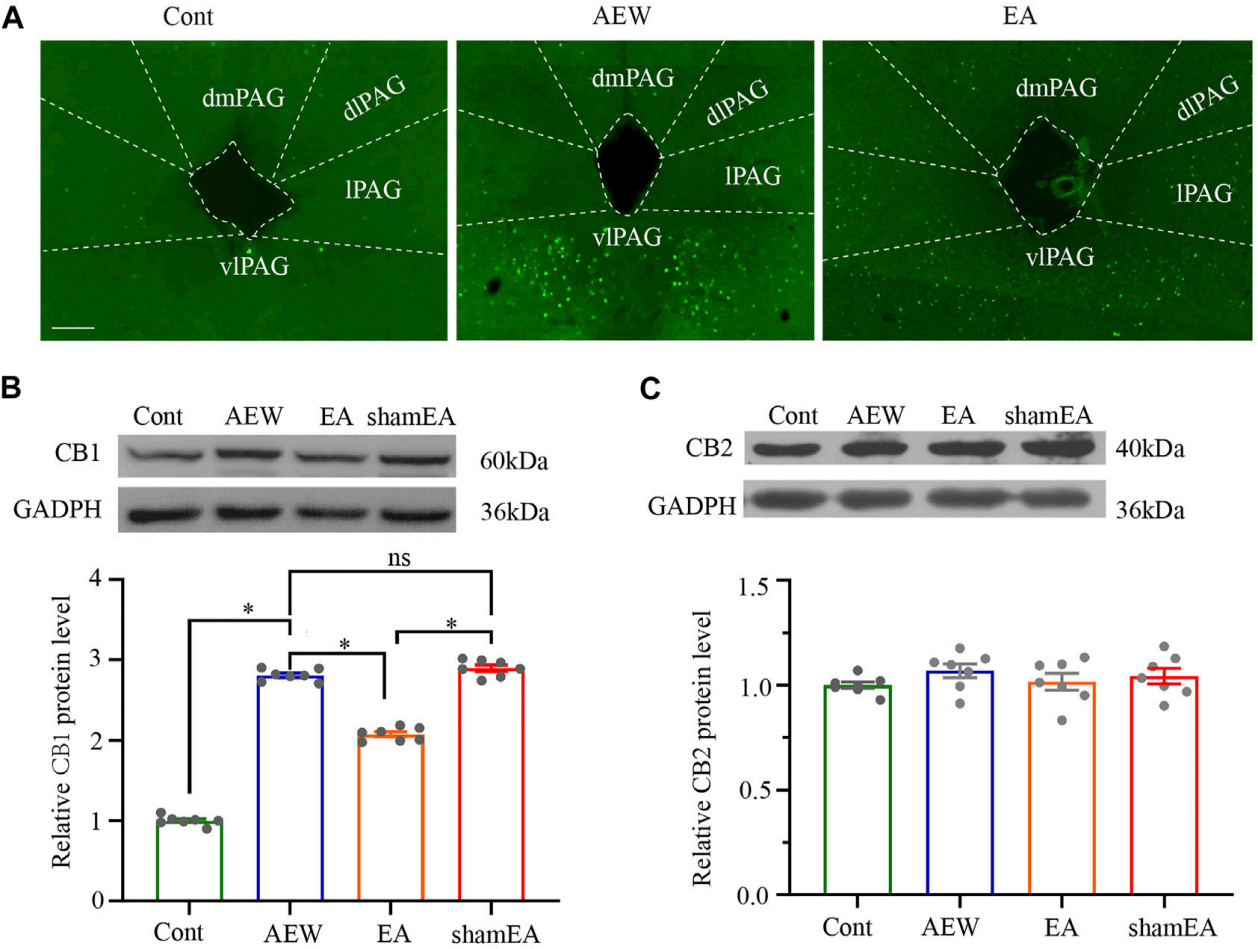
Effect of EA on the expression of CB1 and CB2 receptor protein levels in the vlPAG. **(A)** The expression of c-Fos in the control group, AEW and EA group. **(B)** Representative gel image (upper) and quantification (bottom) of the protein level of CB1 receptors in control, AEW, EA and sham EA groups. **(C)** Representative gel image (upper) and quantification (bottom) of the protein level of CB2 receptors in control, AEW, EA and sham EA groups. GAPDH (36 kDa) was used as a loading control. The protein band at 60 kDa corresponds to the CB1 receptors protein. The protein band at 40 kDa corresponds to the CB2 receptors protein. Data are expressed as means ± SEM (*n* = 6 mice in each group). *p < 0.05.

Considering that CB1 activation in the vlPAG plays a prominent role in analgesia (Maione et al., 2006; Yuan et al., 2018; Zhu et al., 2019), CB1 receptors may modulate pruritus similarly. The c-Fos expression of AEW in the vlPAG was significantly increased compared with that in the vehicle control group (Figure 2A). EA decreased c-Fos expression in the vlPAG compared with the AEW group (Figure 2A), suggesting that EA inhibits itch via reducing the neuronal excitability in the vlPAG. Then, we investigated whether CB1 receptors participated in the antipruritic effect of EA. The CB1 receptor protein level in the vlPAG of AEW group was significantly increased on the 10th day compared with the vehicle control group (*p* < 0.05, Figure 2B). Compared with the AEW group, the expression level of CB1 receptors in vlPAG was significantly reduced by the EA treatment (*p* < 0.05, Figure 2B). The expression of CB1 receptor protein in the vlPAG of the EA group was also significantly lower than that of the sham EA group (*p* < 0.05, Figure 2B). There was no significant difference in the CB2 receptor protein level in the vlPAG among various groups (Figure 2C). EA can significantly reduce the expression of CB1, but not CB2, receptors in the vlPAG, suggesting that CB1 receptors in the vlPAG are involved in the antipruritic effect of EA.

### EA reduces the scratching response *via* activation of CB1 receptors and subsequent inhibition of GRPR and 5-HT

**FIGURE 3:**
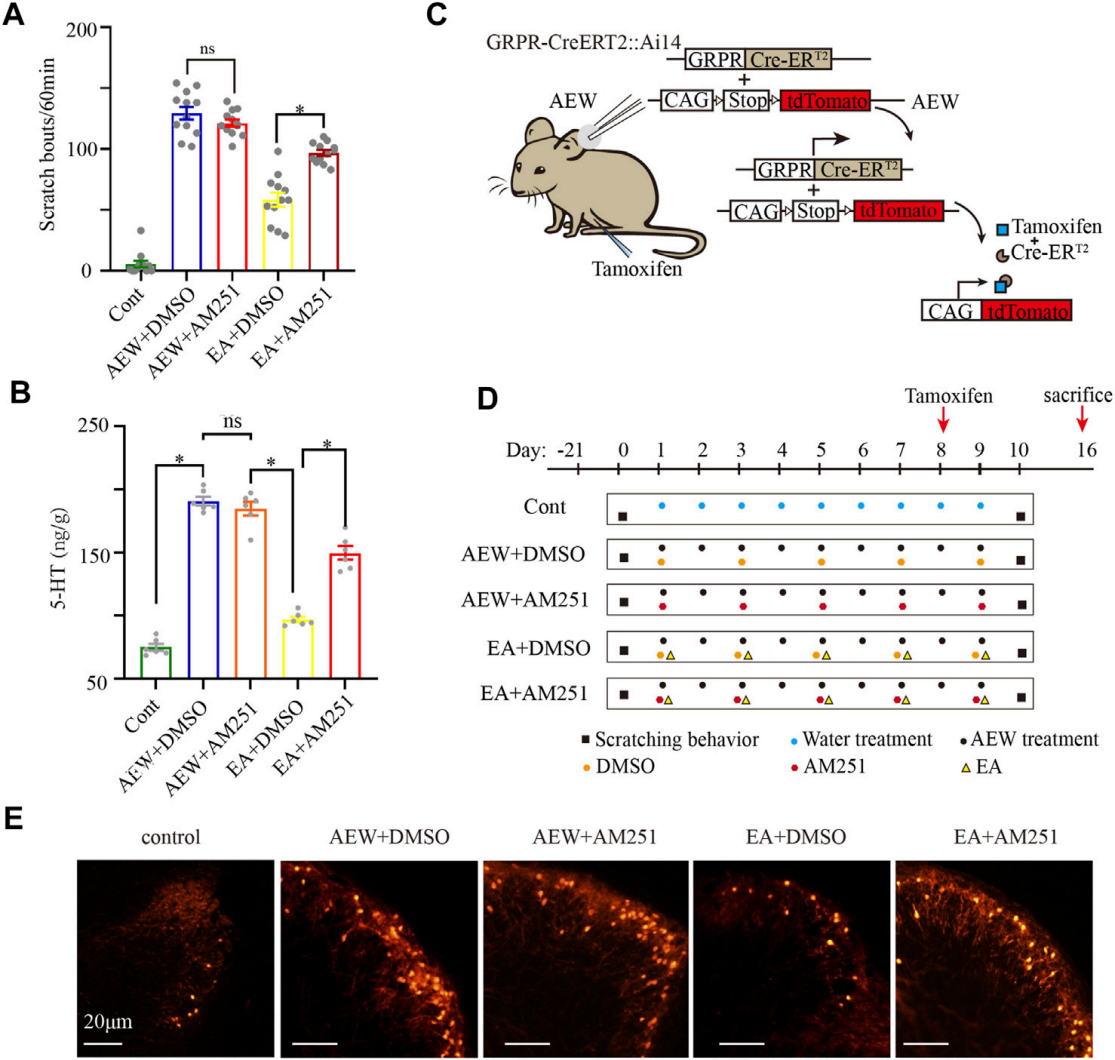
AM251 reverses the antipruritic effect of EA on AEW mice *via* CB1 receptors. **(A)** The scratch bouts in control, AEW+DMSO, AEW+AM251, EA+DMSO and EA+AM251 groups. **(B)** The 5-HT concentration in the medulla oblongata in control, AEW+DMSO, AEW+AM251, EA+DMSO and EA+AM251 groups. **(C,D)** Experimental strategy for AM251 inhibiting the effect of EA on GRPR. **(E)** GRPR expression in the cervical cord in control, AEW+DMSO, AEW+AM251, EA+DMSO and EA+AM251 groups. Scale bar, 20 μm. Data are expressed as means ± SEM (*n* = 12 mice in each group in A, *n* = 6 samples in each group in **(B)**. *p < 0.05.

To further demonstrate the important role of CB1 receptors in the antipruritic effect of EA, mice received systemic injection of AM251, a specific CB1 receptor antagonist, 30 min before each EA treatment. There was no significant difference in the scratching behavior between the AEW+DMSO group and AEW+AM251 group (*p* > 0.05, Figure 3A), suggesting that AM251 itself does not affect itch, which is consistent with previous work (Spradley et al., 2012). However, the antipruritic effect of EA was blocked by administration of AM251 (*p* < 0.05, Figure 3A). 5-HT is a neuromodulator that plays a major role in the descending control of the itch signal processing (Zhao et al., 2014). The 5-HT concentration in the medulla oblongata in AEW+DMSO was significantly increased compared with that in the control group (*p* < 0.05, Figure 3B). AM251 did not block the increase of 5-HT induced by AEW (*p* > 0.05, Figure 3B), but it reversed the inhibitory effect of EA on 5-HT. Compared with EA+DMSO group, the 5-HT concentration in EA+AM251 group was significantly increased (*p* < 0.05, Figure 3B).

GRPR in the cervical cord, an itch-specific receptor, plays a critical role in the transmission of itch information (Liu et al., 2019). The GRPR-iCreERT2:Ai14 mice and tamoxifen were used to quantify the number of GRPR-positive neurons. GRPR-iCreERT2 mice are a class of mice with expression of fusion protein of Cre recombinase and estrogen receptor (ER) ligand-binding region mutant (ERT). Ai14 mice are a class of mice with expression of Cre reporter allele designed with a STOP box flanking loxP to prevent transcription of the CAG promoter driven red fluorescent protein variant (tdTomato). The offspring of these two mouse hybrids GRPR-iCreERT2:Ai14 mice were carried the above gene fragments (Figure 3C). In the absence of Tamoxifen induction, Cre-ERT2 was inactive in cytoplasm. Treatment of AEW induces itch, which results in the expression of GRPR protein and Cre-ERT2 (Figure 3C). Administering tamoxifen immediately on the 8th day of chronic itch (Figure 2D). When Tamoxifen (0.2 mg/g)is induced, 4-OHT (estrogen analogue), the metabolite of Tamoxifen, binds to ERT and induces Cre recombinase activity in the nucleus of GRPR-iCreERT2 cells (Figure 3C). Thus, GRPR + neurons express red fluorescent protein variant (tdTomato). This process of expression red fluorescent protein takes about 8 days (Figure 3D). Mice were sacrificed on the 16th day (8 days after tamoxifen injection to successfully induce GRPR expression, Figures 3C,D). Compared with AEW+DMSO group, the number of GRPR + neurons in the cervical spinal cord of the EA+DMSO group seemed decreased. AEW+AM251 seemed to have no significant effect on the number of GRPR + neurons in the cervical spinal cord compared with that in the AEW+DMSO group. AM251 (EA+AM251) seemed to increase the GRPR protein level in the cervical spinal cord compared with the EA + DMSO group (Figure 3E). It still needs future study to prove more evidence, but these results indicating that EA may reduce itch through CB1 receptors and subsequent inhibition of 5-HT release and GRPR expression.

### CB1 receptors on GABAergic neurons in the vlPAG mediates chronic itch

**FIGURE 4:**
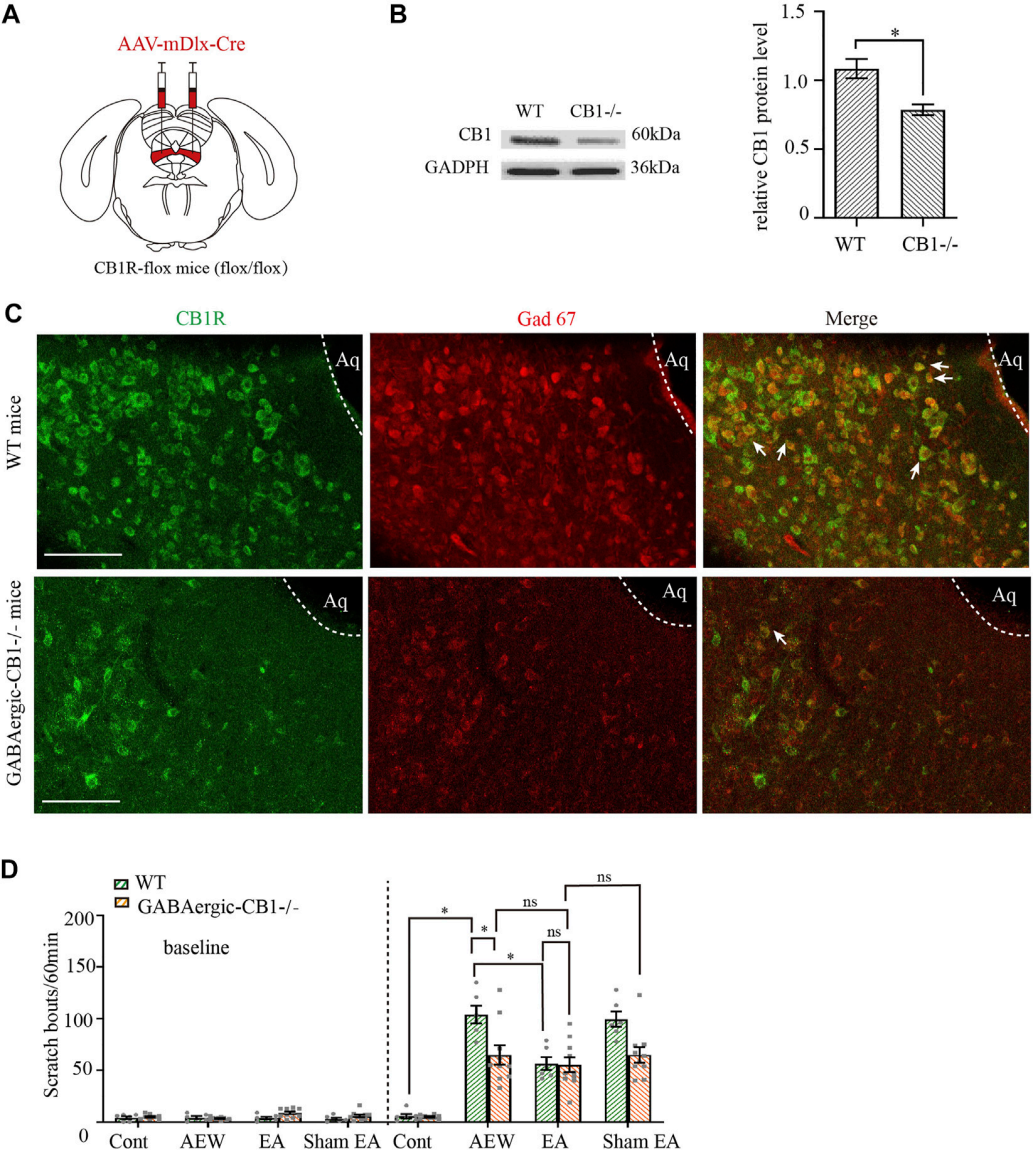
Comparison of the effects of EA on itch in control mice and GABAergic -CB1−/− mice. **(A)** Schematic showing viral targeting of AAV2/9-mDlx-Cre transgenes bilaterally injected into the vlPAG. **(B)** Representative gel image (left) and quantification (right) of the protein level of CB1 receptors in wild-type mice and GABAergic -CB1−/− mice. **(C)** Double staining of CB1 receptors (CB1R, green) and GAD67 mRNA (Gad 67, red) of the vlPAG in wild-type mice and GABAergic-CB1−/− mice. **(D)** The comparison of scratch bouts in wild-type mice and GABAergic-CB1−/− mice. Scale bars: 100 μm. Data are expressed as means ± SEM (*n* = 10 mice in GABAergic-CB1−/− mice, *n* = 6 mice in wild type mice). *p < 0.05.

The activity of GABAergic and glutamatergic neurons in the vlPAG is modulated in an opposing manner during itch-evoked scratching (Samineni et al., 2019). To explore whether CB1 receptors on glutamatergic neurons or GABAergic neurons participate in the antipruritic effect of EA, we used CB1R-flox mice. We injected adeno-associated virus (AAV2/9-mDlx-Cre-WPRE-pA) into the vlPAG of CB1R-flox line mice (GABAergic -CB1−/− mice, Figure 4A) to delete CB1 receptors on GABAergic neurons (Yuan et al., 2018; Zhu et al., 2019), whereas wild-type mice (WT) were used as control. mDlx is a specific promoter of GABAergic neuron. AAV2/9-mDlx-Cre-WPRE-pA can infect GABAergic neurons and express Cre recombinase. The Flox locus in CB1-Flox mice can be activated by Cre in GABAergic neurons to knock out the gene (CB1 receptors) between two Flox. With this virus, CB1 receptors on GABAergic neurons can be specificly knockout.

Both the control mice and GABAergic-CB1−/− mice were divided into the water group, the AEW group, the EA group, and the Sham EA group. CB1 receptors were effectively deleted on GABAergic neurons (Figures 4B,C, *p* < 0.05 in Figure 4C). There was no significant difference in the scratching behavior among the groups of mice before itch induction (*p* > 0.05, Figure 4D). In wild-type mice, the scratching behavior caused by AEW was increased (*p* < 0.05, Figure 4D). Compared with AEW group, the scratching behavior was decreased by EA treatment (*p* < 0.005, Figure 4D). There was no difference between the AEW group and sham EA group (*p* > 0.05, Figure 4D). Ablation of CB1 receptors on GABAergic neurons significantly reduced the scratching behavior in the AEW group compared with the wild-type mice (*p* < 0.05, Figure 4D). After knockout of CB1 receptors on GABAergic neurons, EA and sham EA had no effect on AEW-induced scratching behavior (*p* > 0.05, Figure 4D). Moreover, there was no significant difference in the scratching behavior in EA groups of wild-type mice and GABAergic -CB1−/− mice (*p* > 0.05, Figure 4D). Therefore, these data suggest that CB1 receptors on GABAergic neurons mediates chronic itch.

### CB1 receptors on glutamatergic neurons in the vlPAG are involved in the anti-pruritic effect of EA

**FIGURE 5:**
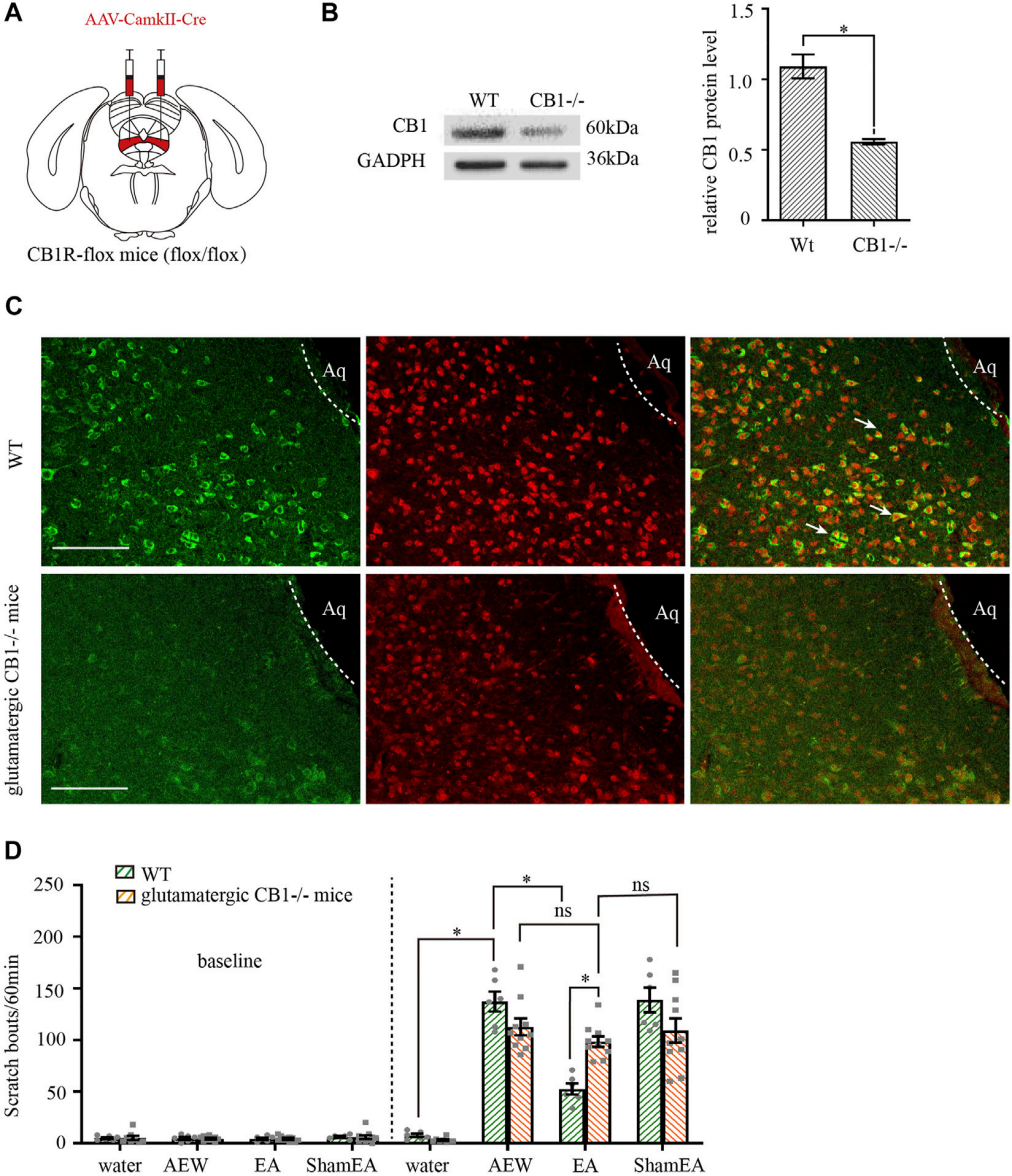
Comparison of the effects of EA on itch in control mice and glutamatergic -CB1−/− mice. **(A)** Schematic showing viral targeting of AAV2/9-CamkII-Cre transgenes bilaterally injected into the vlPAG. **(B)** Representative gel image (left) and quantification (right) of the protein level of CB1 receptors in wild type mice and glutamatergic neurons -CB1−/− mice. **(C)** Double staining of CB1 receptors (CB1R, green) and vglut2 mRNA (vglut2, red) of the vlPAG in wild-type mice and glutamatergic -CB1−/− mice. **(D)** The comparison of scratch bouts in wild-type mice and glutamatergic -CB1−/− mice. Scale bars: 100 μm. Data are expressed as means ± SEM (*n* = 10 mice in glutamatergic neurons -CB1−/− mice, *n* = 6 mice in wild-type mice). *p < 0.05.

We injected adeno-associated virus (AAV2/9-CaMKII-Cre-WPRE-pA) into vlPAG of CB1R-flox line mice (glutamatergic-CB1−/− mice, Figure 5A) to delete CB1 receptors on glutamatergic neurons (Zhu et al., 2019), whereas wild-type mice were used as control. CaMKII is a specific promoter of glutamatergic neuron. AAV2/9-mDlx-Cre-WPRE-pA can induce expression of Cre recombinase glutamatergic neurons. AAV2/9- CaMKII -Cre-WPRE-pA can infect glutamatergic neurons and express Cre recombinase. The Flox locus in CB1-Flox mice can be activated by Cre recombinase in glutamatergic neurons to knock out the gene (CB1 receptors) between two Flox. With this virus, CB1 receptors on glutamatergic neurons can be specificly knockout.

Both the control mice and glutamatergic-CB1−/− mice were divided into the water group, the AEW group, the EA group and the sham EA group. CB1 receptors were effectively deleted on glutamatergic neurons (Figures 5B,C, *p* < 0.05 in Figure 5B). There was no significant difference in the scratching behavior among the groups of mice before itch induction (*p* > 0.05, Figure 5D). In wild-type mice, the scratching behavior caused by AEW was increased (*p* < 0.05, Figure 5D). Compared with AEW group, the scratching behavior was decreased after EA treatment (*p* < 0.05, Figure 5D). There was no difference between AEW group and sham EA group (*p* > 0.05, Figure 5D). Knockout of CB1 receptors on glutamatergic neurons did not affect the scratching behavior in AEW group and sham EA group compared with that in wild-type mice (*p* > 0.05, Figure 5D). However, after knockout of CB1 receptors on glutamatergic neurons, EA and sham EA had no effect on AEW-induced scratching behavior (*p* > 0.05, Figure 5D). Compared with the EA group in wild-mice, the scratching behavior of EA group in glutamatergic -CB1−/− mice was increased (*p* < 0.05, Figure 5D), suggesting that CB1 receptors on glutamatergic neurons mediates the antipruritic effect of EA.

### CB1 receptors are involved in the EA effect on the 5-HT concentration in the medulla oblongata

**FIGURE 6:**
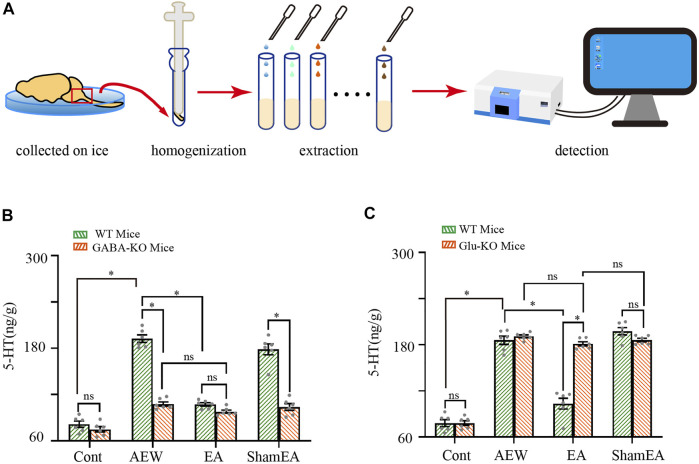
Comparison of the effects of EA on 5-HT in wild-type mice and CB1-KO mice. **(A)** Schematic showing the measurement of 5-HT. **(B)** Summary data show the effect of EA on the 5-HT concentration in the medulla of wild type mice and GABA -CB1−/− mice. (*n* = 10 mice in CB1-KO mice, *n* = 6 mice in wild-type mice). **(C)** Summary data show the effect of EA on the 5-HT concentration in the medulla of wild-type mice and glutamatergic -CB1−/− mice. Data are expressed as means ± SEM (*n* = 10 mice in CB1-KO mice, *n* = 6 mice in wild type mice). *p < 0.05.

Lastly, we determined whether CB1 receptors are involved in the effect of EA on the 5-HT concentration in the medulla oblongata. The experimental protocol was shown in Figure 6A. In wild-type mice, the 5-HT level in the medulla oblongata was increased by AEW (*p* < 0.05, Figures 6B,C). Compared with the AEW group, the 5-HT level in the medulla oblongata was decreased after EA treatment (*p* < 0.05, Figures 6B,C). Knockout of CB1 receptors on GABAergic neurons reduced the 5-HT level in the medulla oblongata in the AEW group compared with wild-type mice (*p* < 0.05, Figure 6B). After the knockout of CB1 receptors on GABAergic neurons, the 5-HT concentration did not differ significantly between EA or sham EA groups and the AEW group (*p* > 0.05, Figure 6B). Moreover, there was no difference between the EA group in GABAergic-CB1−/− mice and wild-type mice (*p* > 0.05, Figure 6B). Knockout of CB1 receptors on glutamatergic neurons did not affect the increase of 5-HT concentration in the AEW group (*p* > 0.05, Figure 6C). However, the 5-HT level in the medulla oblongata in the EA group in glutamatergic-CB1−/− mice was increased compared with wild-type mice, suggesting that CB1 receptors on glutamatergic neurons mediate the inhibitory effect of EA on the increasing of 5-HT in the medulla oblongata.

## Discussion

EA has been commonly used for treating itch, but the mechanisms involved in the antipruritic effect of EA are unclear. In this study, we demonstrated that EA treatment (100 Hz, 3 mA) at Quchi and Hegu points was the best stimulation parameters of EA on inhibiting scratching behavior. EA significantly reduced the 5-HT levels and the number of GRPR-positive neurons in the spinal dorsal horn as well as the expression of CB1 receptors in the vlPAG. Furthermore, knockout of CB1 receptors on GABAergic neurons resulted in reduction of scratching behavior and 5-HT levels in the medulla oblongata and knockout of CB1 receptors on glutamatergic neurons blocked the antipruritic effects of EA and the inhibitory effect of EA on 5-HT concentrations in the medulla oblongata. Therefore, our study provides new information that EA treatment reduces chronic itch by activation of CB1 receptors on glutamatergic neurons and downregulating CB1 receptors on GABAergic neurons in the vlPAG. Via this differential effect, EA inhibits the release of 5-HT from the medulla oblongata to the GRPR–positive neurons in the spinal cord.

Previous studies suggest that high-frequency EA (100 Hz) is an effective antipruritic treatment (Li et al., 2021). Therefore, by keeping the EA frequency at 100Hz, we determined what intensity and acupoint loci are most effective. We found that the high intensity (3 mA) was better than the low intensity (1 mA) in reducing spontaneous scratching behavior. Quchi (LI11), Zusanli (ST36), Xuehai (SP10), and Hegu (LI14) (Yin et al., 2008) are important acupoints for treating itch. Our results showed that the effect of EA at Quchi and Hegu was better than Zusanli and Xuehai. The two acupoints Quchi and Hegu are located in the same spinal cord segment as the cervical skin lesions, which may explain their efficacy.

Despite the abundant evidence supporting the role of CB1 receptors in the vlPAG in EA analgesia (Yuan et al., 2018; Zhu et al., 2019), it is uncertain whether the CB1 receptors play a role in the EA antipruritic effect. We showed that the expression of CB1 receptors in vlPAG was significantly increased in the AEW group, and EA significantly reduced the CB1 receptor level in the vlPAG. These results suggest that EA relieve chronic itch mainly by the down-regulation of the CB1 receptors in the vlPAG. Also, we found that the CB1 receptors antagonist AM251 reversed the effect of EA on chronic itch.

GRPR is crucially involved in itch transmission at the spinal level (Sun and Chen, 2007). Activation of GRPR neurons results in itch. At the spinal level, both direct excitation of spinal GRPR neurons by 5-HT and indirect activation of GRPR neurons by disinhibition mechanism have been demonstrated (Zhao et al., 2014; Huang et al., 2018). We found that 5-HT levels and the expression of GRPR were increased by AEW but decreased by EA. Furthermore, the effect of EA on 5-HT and GRPR was blocked by AM251, suggesting that EA decreased 5-HT concentrations in the medulla oblongata and inhibited activation of GRPR *via* CB1 receptors in the vlPAG.

Cannabinoid CB1 receptors are present at axon terminals of both glutamatergic and GABAergic neurons. Activation of CB1 receptors inhibits the presynaptic release of both GABA and glutamate (Freund et al., 2003). It has been reported that activation of GABAergic neurons or inhibition of glutamatergic neurons in the vlPAG results in attenuation of scratching in both acute and chronic pruritis (Samineni et al., 2019). To distinguish the different roles of CB1 receptors on glutamatergic neurons and GABAergic neurons in the EA effect, we studied conditional knockdown of CB1 receptors in the antipruritic effect of EA. We found that knockout of CB1 receptors on glutamatergic neurons blocked the antipruritic effects of EA and inhibitory effect of EA on 5-HT levels in medulla oblongata. Conditional knockout of CB1 receptors on GABAergic neurons significantly decreased chronic itch and 5-HT levels in medulla oblongata. Since knockout of CB1 receptors on GABAergic neurons mimic the antipruritic effect of EA on AEW treated wild mice, it suggested EA may downregulated CB1 receptors on GABAergic neurons. As the CB1 receptors on GABAergic terminals may reduce GABA release (Fu and Longhurst, 2009; Yuan et al., 2018), the absent of CB1 receptors on GABAergic neurons in the vlPAG may promote GABA release, thus resulting in activation of GABAergic neurons. It was hypothesized that GABAergic neurons have an inhibitory effect on vlPAG glutamatergic neurons (Yin et al., 2020). EA may activate GABAergic neurons and then inhibit glutamatergic neurons, thus decreasing the content of 5-HT in medulla oblongata, and weakening activation of GRPR positive neurons in spinal cord, thus relieving itch.

**FIGURE 7:**
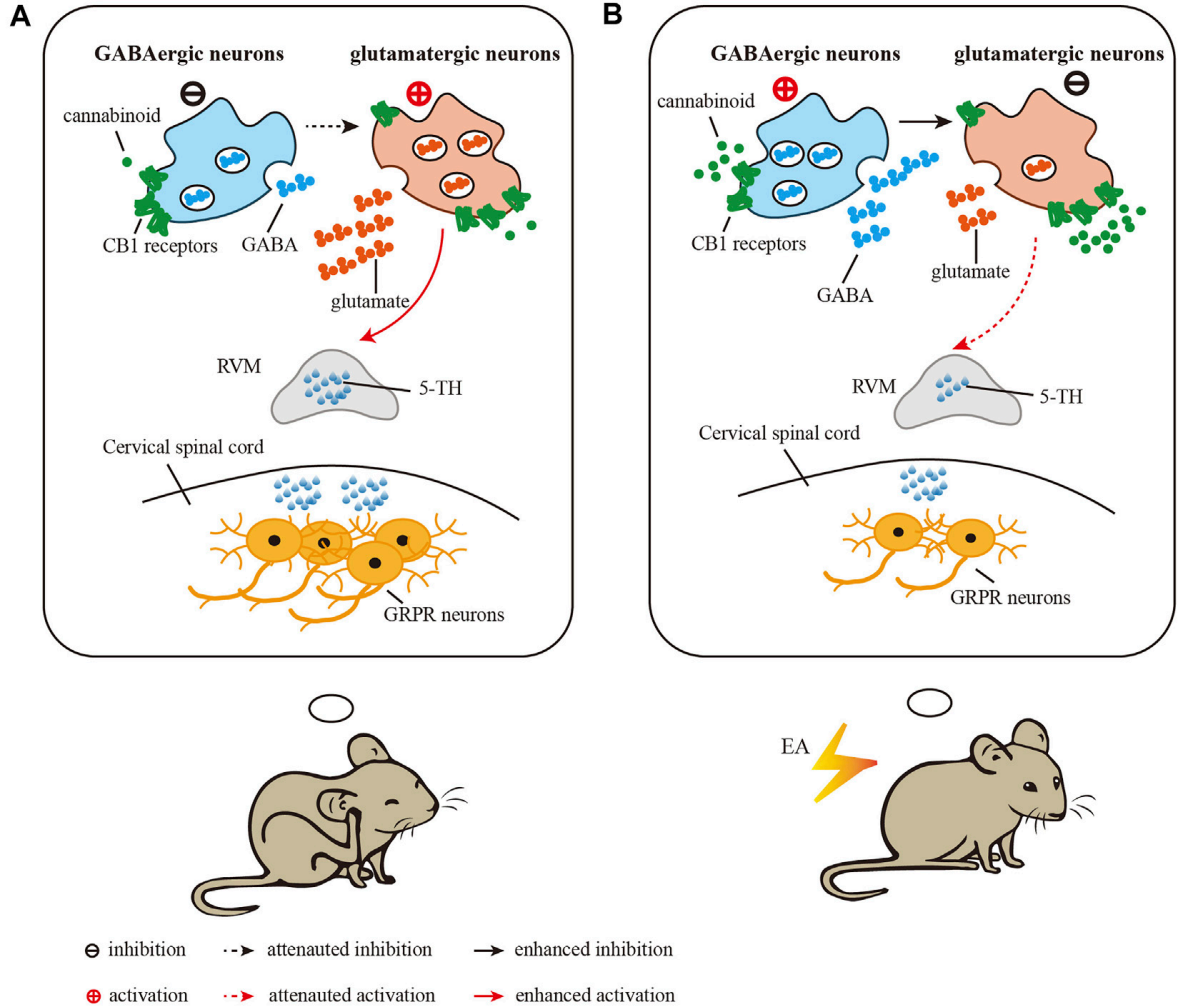
Diagram showing the role of CB1 receptors in the vlPAG in the inhibitory effect of EA on chronic itch. **(A)** in the vlPAG after induction of chronic itch, glutamatergic neurons are activated and GABAergic neurons are inhibited, which activate RVM neurons to increase the level of 5-HT and the expression of GRPR in the spinal cord. **(B)** EA treatment results in activation of GABAergic neurons to inhibit glutamatergic neurons in the vlPAG, thus attenuating the activation of RVM neurons and the level of 5-HT and the expression of GRPR in the spinal cord. EA promots the release of endogenous cannabinoids, which target CB1 receptors on glutamatergic terminals to inhibit neuronal activity. Through these two mechanisms, EA reduces the chronic itch.

Previous studies showed that EA may promoted the release of endogenous cannabinoid in the midbrain (Yuan et al., 2018). Endogenous cannabinoid acts as a retrograde messenger to activate presynaptic CB1 receptors located on the glutamatergic and GABAergic terminals to inhibit GABA and glutamate release. The regulation of EA on CB1 on these two types of neurons is different. It has been reported that EA did not modulate the expression of CB1 receptors on glutamatergic neurons (Yuan et al., 2018). Our results showed EA down-regulated the expression of CB1 receptors. Therefore, the expression of CB1 receptors on GABAergic neurons was down-regulated by EA. The decrease of CB1 receptors weakened the inhibition produced by endogenous cannabinoid of GABAergic neurons, thus resulting in release of GABA and then inhibiting glutamatergic neurons (Figure 7). On the other hand, endogenous cannabinoid promoted by EA target CB1 receptors localized on glutamatergic resulted in inhibition of glutamatergic neurons. As glutamate neurons in the vlPAG are output neurons that project to the RVM (Yin et al., 2020), EA may inhibit glutamatergic neurons via CB1 receptors to reduce the content of 5-HT in the RVM and cervical spinal cord, thereby attenuating the activation of GRPR neurons in the cervical spinal cord and inhibiting chronic itch (Figure 7). In summary, EA down-regulated CB1 receptor on GABAergic neurons to increase the release of GABA, which resulting in the inhibition of glutamatergic neurons. Meanwhile, EA acted CB1 receptors on glutamatergic neurons to inhibit glutamatergic neurons by promoting the release of endogenous cannabinoid. Besides glutamate and GABA, there may be other neuropeptides involving in treatment of EA. For example, besides glutamate and GABA, there may be other neuropeptides involving in treatment of EA. For example, it has been reported that the orexin neurons from hypothalamus can modulate the endocannabinoid system in the PAG during EA (Chen et al., 2018), and orexin 1 receptor-initiated endocannabinoid/CB1 signaling in the mouse PAG (Lee et al., 2016). Therefore, the mechanism of EA reduces chronic itch via cannabinoid CB1 receptors in the vlPAG is complex and needs further study. The mechanism of EA reduces chronic itch *via* cannabinoid CB1 receptors in the vlPAG is complex and needs further study.

There were some limitations of the study. It still needs future study to prove that EA may reduce itch through inhibition of 5-HT release and GRPR expression. In this study, GRPR-iCreERT2:Ai14 mice were used to evaluate the GRPR expression. However, this experiment may not fully support to represented the GRPR expression. There is one possible that both the number of fluorescent cells and fluorescent intensity represent the protein expression level of GPRP. The more number and the brighter fluorescence intensity means higher protein expression level GRPR. It is better to detect GRPR protein level *via* WB. However, antibodies for GRPR with good specificity have not been found yet. Therefore, we have to use the number of GRPR + neurons to represcent GRPR expression as a compromise.

In this study, we showed for the first time that high frequency (100Hz) and high intensity (3 mA) of EA at the same spinal segment where itch was induced are the most effective on dry skin-induced chronic itch. This information could guide the clinical treatment of chronic itch and improve the therapeutic effect of acupuncture. Furthermore, we showed that the endogenous cannabinoid system is involved in the effect of EA on chronic itch. EA mainly inhibits glutamatergic neurons through CB1 receptors in the vlPAG. As a result, EA reduces the content of 5-HT in the RVM, thus inhibiting GRPR signaling and chronic itch. This new information advances our knowledge on the mechanisms underlying the therapeutic effect of EA on chronic itch and provides further rationale for using EA to for treating chronic itch.

## Data Availability

The original contributions presented in the study are included in the article/supplementary materials, further inquiries can be directed to the corresponding authors.

## References

[B1] Akiyama T. Carstens E. (2013). Neural processing of itch. Neuroscience 250, 697–714. 10.1016/j.neuroscience.2013.07.035 23891755 PMC3772667

[B2] Akiyama T. Carstens M. I. Ikoma A. Cevikbas F. Steinhoff M. Carstens E. (2012). Mouse model of touch-evoked itch (alloknesis). J. Invest. Dermatol. 132 (7), 1886–1891. 10.1038/jid.2012.52 22418875 PMC3375351

[B3] Akiyama T. Nguyen T. Curtis E. Nishida K. Devireddy J. Delahanty J. (2015). A central role for spinal dorsal horn neurons that express neurokinin-1 receptors in chronic itch. Pain 156 (7), 1240–1246. 10.1097/j.pain.0000000000000172 25830923 PMC4474752

[B4] Bilir K. A. Anli G. Ozkan E. Gunduz O. Ulugol A. (2018). Involvement of spinal cannabinoid receptors in the antipruritic effects of WIN 55, 212-2, a cannabinoid receptor agonist. Clin. Exp. Dermatol. 43 (5), 553–558. 10.1111/ced.13398 29424035

[B5] Bourane S. Duan B. Koch S. C. Dalet A. Britz O. Garcia-Campmany L. (2015). Gate control of mechanical itch by a subpopulation of spinal cord interneurons. Science 350 (6260), 550–554. 10.1126/science.aac8653 26516282 PMC4700934

[B6] Chen Y. H. Lee H. J. Lee M. T. Wu Y. T. Lee Y. H. Hwang L. L. (2018). Median nerve stimulation induces analgesia via orexin-initiated endocannabinoid disinhibition in the periaqueductal gray. Proc. Natl. Acad. Sci. U. S. A. 115 (45), E10720–E10729. 10.1073/pnas.1807991115 30348772 PMC6233149

[B7] Darmani N. A. Pandya D. K. (2000). Involvement of other neurotransmitters in behaviors induced by the cannabinoid CB1 receptor antagonist SR 141716A in naive mice. J. Neural Transm. 107 (8-9), 931–945. 10.1007/s007020070043 11041273

[B8] Duo L. J. (1987). Electrical needle therapy of uremic pruritus. Nephron 47 (3), 179–183. 10.1159/000184487 3500424

[B9] Freund T. F. Katona I. Piomelli D. (2003). Role of endogenous cannabinoids in synaptic signaling. Physiol. Rev. 83 (3), 1017–1066. 10.1152/physrev.00004.2003 12843414

[B10] Fu L. W. Longhurst J. C. (2009). Electroacupuncture modulates vlPAG release of GABA through presynaptic cannabinoid CB1 receptors. J. Appl. Physiol. 106 (6), 1800–1809. 10.1152/japplphysiol.91648.2008 19359606 PMC2692780

[B11] Huang J. Polgar E. Solinski H. J. Mishra S. K. Tseng P. Y. Iwagaki N. (2018). Author Correction: circuit dissection of the role of somatostatin in itch and pain. Nat. Neurosci. 21 (6), 894. 10.1038/s41593-018-0149-6 29674654

[B12] Jung D. L. Lee S. D. Choi I. H. Na H. S. Hong S. U. (2014). Effects of electroacupuncture on capsaicin-induced model of atopic dermatitis in rats. J. Dermatol. Sci. 74 (1), 23–30. 10.1016/j.jdermsci.2013.11.015 24418195

[B13] Kan B. H. Yu J. C. Zhao L. Zhao J. Li Z. Suo Y. R. (2018). Acupuncture improves dendritic structure and spatial learning and memory ability of Alzheimer's disease mice. Neural Regen. Res. 13 (8), 1390–1395. 10.4103/1673-5374.235292 30106051 PMC6108219

[B14] Kim K. H. Lee M. S. Choi S. M. (2010). Acupuncture for treating uremic pruritus in patients with end-stage renal disease: a systematic review. J. Pain Symptom Manage. 40 (1), 117–125. 10.1016/j.jpainsymman.2009.11.325 21796811

[B15] LaMotte R. H. Dong X. Ringkamp M. (2014). Sensory neurons and circuits mediating itch. Nat. Rev. Neurosci. 15 (1), 19–31. 10.1038/nrn3641 24356071 PMC4096293

[B42] Lee H. J. Chang L. Y. Ho Y. C. Teng S. F. Hwang L. L. Mackie K. (2016). Stress induces analgesia via orexin 1 receptor-initiated endocannabinoid/CB1 signaling in the mouse periaqueductal gray. Neuropharmacology 105, 577–586. 10.1016/j.neuropharm.2016.02.018 26907809 PMC8081448

[B16] Li H. P. Wang X. Y. Chen C. Li J. J. Yu C. Lin L. X. (2021). 100 Hz electroacupuncture alleviated chronic itch and GRPR expression through activation of kappa opioid receptors in spinal dorsal horn. Front. Neurosci. 15, 625471. 10.3389/fnins.2021.625471 33664646 PMC7921323

[B17] Lim S. (2010). WHO standard acupuncture point locations. Evid. Based. Complement. Altern. Med. 7 (2), 167–168. 10.1093/ecam/nep006 PMC2862941 19204011

[B18] Liu M. Z. Chen X. J. Liang T. Y. Li Q. Wang M. Zhang X. Y. (2019). Synaptic control of spinal GRPR(+) neurons by local and long-range inhibitory inputs. Proc. Natl. Acad. Sci. U. S. A. 116, 27011–27017. 10.1073/pnas.1905658116 PMC6936532 31806757

[B19] Maione S. Palazzo E. de Novellis V. Stella L. Leyva J. Rossi F. (1998). Metabotropic glutamate receptors modulate serotonin release in the rat periaqueductal gray matter. Naunyn. Schmiedeb. Arch. Pharmacol. 358 (4), 411–417. 10.1007/pl00005272 9826062

[B20] Maione S. Bisogno T. de Novellis V. Palazzo E. Cristino L. Valenti M. (2006). Elevation of endocannabinoid levels in the ventrolateral periaqueductal grey through inhibition of fatty acid amide hydrolase affects descending nociceptive pathways via both cannabinoid receptor type 1 and transient receptor potential vanilloid type-1 receptors. J. Pharmacol. Exp. Ther. 316 (3), 969–982. 10.1124/jpet.105.093286 16284279

[B21] Metz M. Grundmann S. Stander S. (2011). Pruritus: an overview of current concepts. Vet. Dermatol. (2), 121–131. 10.1111/j.1365-3164.2010.00945.x 21251097

[B22] Nojima H. Carstens M. I. Carstens E. (2003). c-fos expression in superficial dorsal horn of cervical spinal cord associated with spontaneous scratching in rats with dry skin. Neurosci. Lett. 347 (1), 62–64. 10.1016/s0304-3940(03)00609-8 12865142

[B23] Odan M. Ishizuka N. Hiramatsu Y. Inagaki M. Hashizume H. Fujii Y. (2012). Discovery of S-777469: an orally available CB2 agonist as an antipruritic agent. Bioorg. Med. Chem. Lett. 22 (8), 2803–2806. 10.1016/j.bmcl.2012.02.072 22444677

[B24] Samineni V. K. Grajales-Reyes J. G. Sundaram S. S. Yoo J. J. Gereau R. W. t. (2019). Cell type-specific modulation of sensory and affective components of itch in the periaqueductal gray. Nat. Commun. 10 (1), 4356. 10.1038/s41467-019-12316-0 31554789 PMC6761157

[B25] Schlosburg J. E. Boger D. L. Cravatt B. F. Lichtman A. H. (2009). Endocannabinoid modulation of scratching response in an acute allergenic model: a new prospective neural therapeutic target for pruritus. J. Pharmacol. Exp. Ther. 329 (1), 314–323. 10.1124/jpet.108.150136 19168707 PMC2670585

[B26] Schlosburg J. E. O'Neal S. T. Conrad D. H. Lichtman A. H. (2011). CB1 receptors mediate rimonabant-induced pruritic responses in mice: investigation of locus of action. Psychopharmacol. Berl. 216 (3), 323–331. 10.1007/s00213-011-2224-5 PMC3606913 21340468

[B27] Sharma A. Haseeb A. Abuzar S. (2006). Screening of field pea (Pisum sativum) selections for their reactions to root-knot nematode (*Meloidogyne incognita*). J. Zhejiang Univ. Sci. B 7 (3), 209–214. 10.1631/jzus.2006.B0209 16502508 PMC1419065

[B28] Spradley J. M. Davoodi A. Gee L. B. Carstens M. I. Carstens E. (2012). Differences in peripheral endocannabinoid modulation of scratching behavior in facial vs. spinally-innervated skin. Neuropharmacology 63 (4), 743–749. 10.1016/j.neuropharm.2012.05.032 22683515 PMC3394407

[B29] Sun Y. G. Chen Z. F. (2007). A gastrin-releasing peptide receptor mediates the itch sensation in the spinal cord. Nature 448 (7154), 700–703. 10.1038/nature06029 17653196

[B30] Tjen A. L. S. C. Li P. Longhurst J. C. (2009). Processing cardiovascular information in the vlPAG during electroacupuncture in rats: roles of endocannabinoids and GABA. J. Appl. Physiol. 106 (6), 1793–1799. 10.1152/japplphysiol.00142.2009 19325030 PMC2692771

[B31] Tosun N. C. Gunduz O. Ulugol A. (2015). Attenuation of serotonin-induced itch responses by inhibition of endocannabinoid degradative enzymes, fatty acid amide hydrolase and monoacylglycerol lipase. J. Neural Transm. 122 (3), 363–367. 10.1007/s00702-014-1251-x 24915981

[B32] Wang Y. Fu Y. Zhang L. Fu J. Li B. Zhao L. (2019). Acupuncture needling, electroacupuncture, and fire needling improve imiquimod-induced psoriasis-like skin lesions through reducing local inflammatory responses. Evid. Based. Complement. Altern. Med. 2019, 4706865. 10.1155/2019/4706865 PMC6699296 31467575

[B33] Wu C. X. Feng Y. H. Yang L. Zhan Z. L. Xu X. H. Hu X. Y. (2018). Electroacupuncture exerts neuroprotective effects on ischemia/reperfusion injury in JNK knockout mice: the underlying mechanism. Neural Regen. Res. 13 (9), 1594–1601. 10.4103/1673-5374.235294 30127120 PMC6126120

[B34] Yin C. S. Jeong H. S. Park H. J. Baik Y. Yoon M. H. Choi C. B. (2008). A proposed transpositional acupoint system in a mouse and rat model. Res. Vet. Sci. 84 (2), 159–165. 10.1016/j.rvsc.2007.04.004 17559895

[B35] Yin J. B. Liang S. H. Li F. Zhao W. J. Bai Y. Sun Y. (2020). dmPFC-vlPAG projection neurons contribute to pain threshold maintenance and antianxiety behaviors. J. Clin. Invest. 130 (12), 6555–6570. 10.1172/JCI127607 32841213 PMC7685740

[B36] Yosipovitch G. Greaves M. W. Schmelz M. (2003). Itch. Lancet 361 (9358), 690–694. 10.1016/S0140-6736(03)12570-6 12606187

[B37] Yuan X. C. Zhu B. Jing X. H. Xiong L. Z. Wu C. H. Gao F. (2018). Electroacupuncture potentiates cannabinoid receptor-mediated descending inhibitory control in a mouse model of knee osteoarthritis. Front. Mol. Neurosci. 11, 112. 10.3389/fnmol.2018.00112 29681797 PMC5897736

[B38] Zhang X. H. Li F. F. Qi Y. Ming C. R. Li Y. Pan S. T. (2020a). Electroacupuncture improves cutaneous allergic reaction by inhibiting degranulation of intrape-ritoneal mast cells, MAPK signaling and inflammatory factor levels in urticaria rats. Zhen Ci Yan Jiu 45 (4), 299–304. 10.13702/j.1000-0607.190263 32333535

[B39] Zhang X. H. Ma T. M. Ming C. R. Wang L. Chen Y. R. Pan S. T. (2020b). Effect of electroacupuncture on expressions of Lyn and Syk in mast cells of subcutaneous loose connective tissue in rats with urticarial. Zhongguo Zhen Jiu 40 (7), 765–770. 10.13703/j.0255-2930.20191023-k0005 32648402

[B40] Zhao Z. Q. Liu X. Y. Jeffry J. Karunarathne W. K. Li J. L. Munanairi A. (2014). Descending control of itch transmission by the serotonergic system via 5-HT1A-facilitated GRP-GRPR signaling. Neuron 84 (4), 821–834. 10.1016/j.neuron.2014.10.003 25453842 PMC4254557

[B41] Zhu H. Xiang H. C. Li H. P. Lin L. X. Hu X. F. Zhang H. (2019). Inhibition of GABAergic neurons and excitation of glutamatergic neurons in the ventrolateral periaqueductal gray participate in electroacupuncture analgesia mediated by cannabinoid receptor. Front. Neurosci. 13, 484. 10.3389/fnins.2019.00484 31156369 PMC6533898

